# Supplementary material for: Test-System for Bacteria Sensing Based on Peroxidase-Like Activity of Inkjet-Printed Magnetite Nanoparticles
Source: Nanomaterials (Basel). 2020 Feb 12;10(2):313. doi: 10.3390/nano10020313 (PMC7075215; doi:10.3390/nano10020313)
Supplement: Supplementary file 1 [file nanomaterials-10-00313-s001.pdf]

Article

Test-System for Bacteria Sensing Based on Peroxidase-Like Activity of Inkjet-Printed Magnetite Nanoparticles

Maxim Zakharzhevskii, Andrey S. Drozdov *, Denis S. Kolchanov, Liubov Shkodenko and Vladimir V. Vinogradov *

Laboratory of Solution Chemistry of Advanced Materials and Technologies, ITMO University, 197101 St. Petersburg, Russia; maxim_z@scamt-itmo.ru (M.Z.); kolchanov@scamt-itmo.ru (D.S.K.); Shkodenko@scamt-itmo.ru (L.S.)

* Correspondence: drozdov@scamt-itmo.ru (A.S.D.); vinogradov@scamt-itmo.ru (V.V.V.)

Received: 07 January 2020; Accepted: 08 February 2020; Published: date

Supplementary Figures

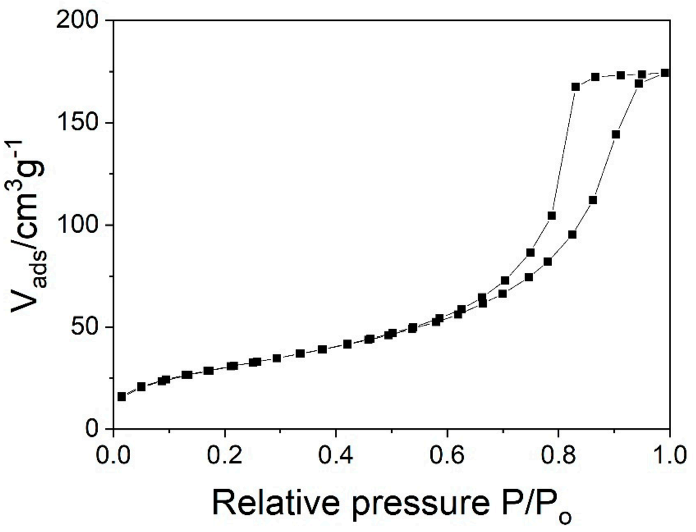

Figure S1. Low-temperature nitrogen sorbtion-desorbtion isotherm of magnetite xerogel,.

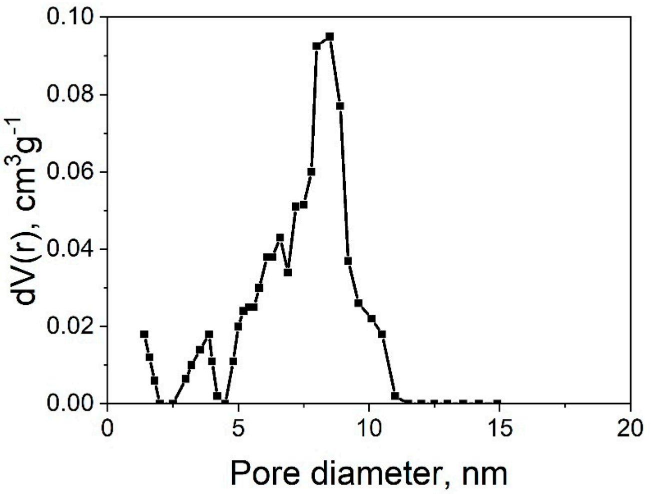

Figure S2. Pore diameter distribution of magnetite xerogel calculated by BJH method.

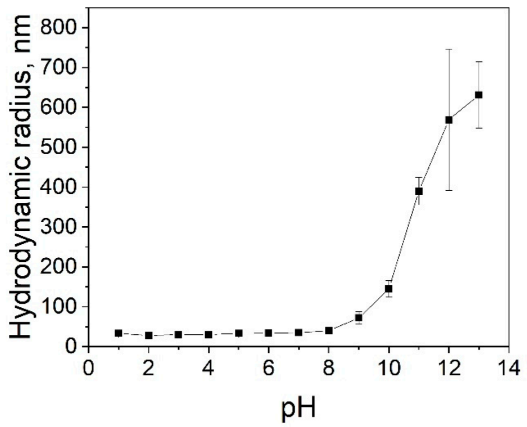

Figure S3. Hydrodynamic radius of MNPs as a function of pH.

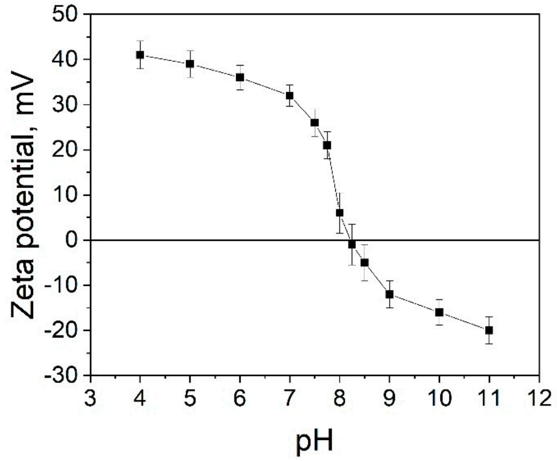

Figure S4. Zeta potential of MNPs as a function of pH.

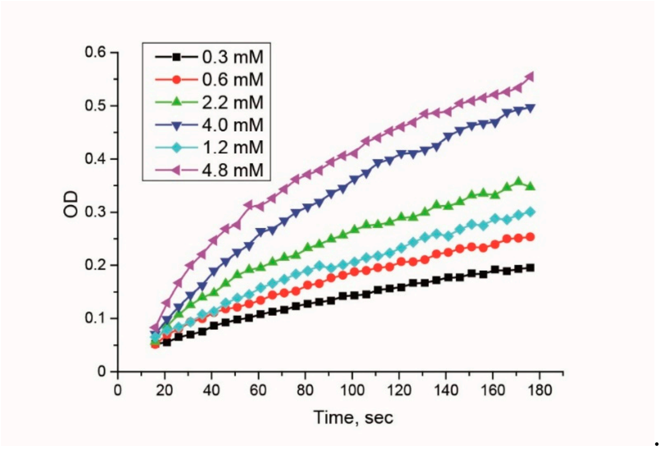

Figure S5. ABTS oxidation kinetics measured at various concentrations of substrate.

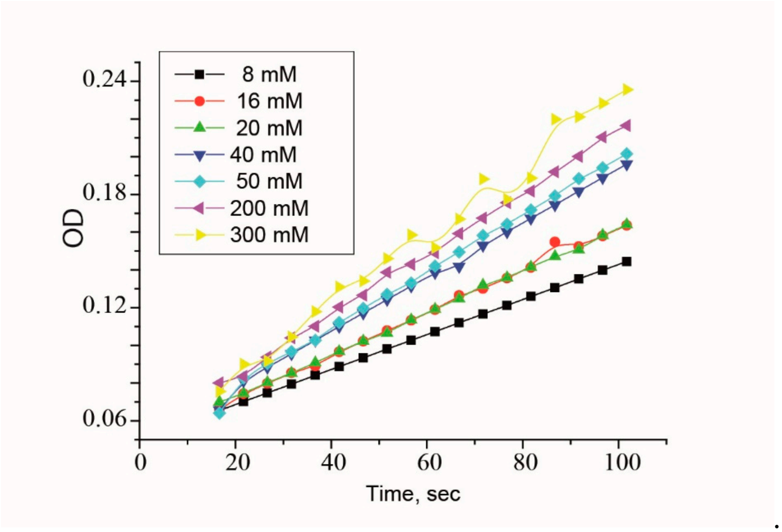

Figure S6. ABTS oxidation kinetics measured at various concentrations of hydrogen peroxide.

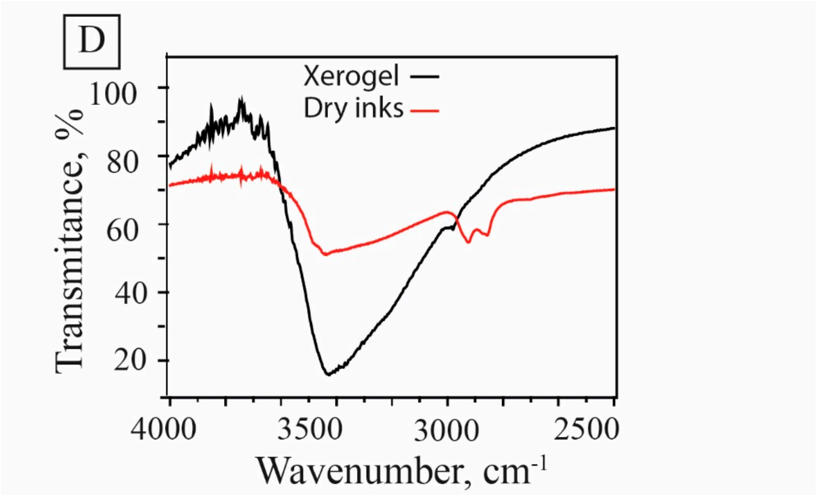

Figure S7. ATR-IR spectrum of xerogels.

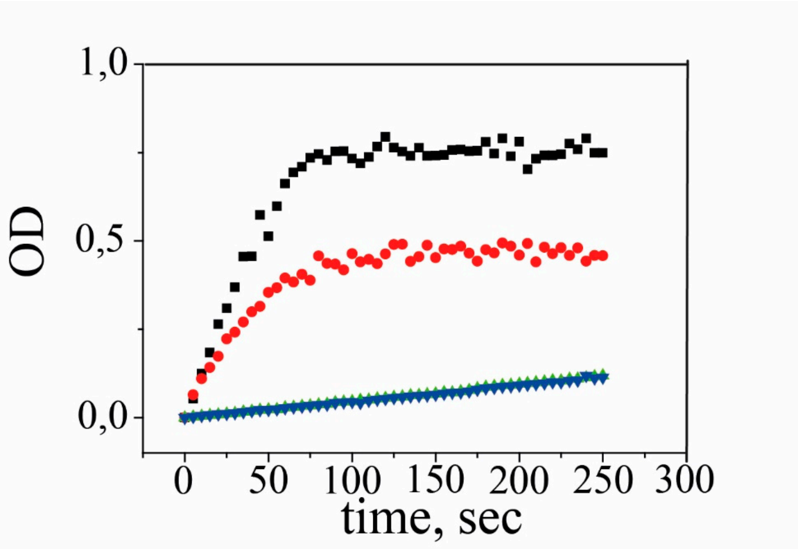

Figure S8. Comparative analysis of peroxidase activity hydrosol (black line), ink (red), xerogel (blue), dry ink (green).

Table 1. Literature data analysis.

**Nanoparticle**	**ABTS**		
	**K_M (mmol/L)**	**V_{max} (10^{-7} M/s)**	**k_{cat} (s^{-1})**
Pristine MNPs	1.22	43,9	365,83
Mb–MNPs[1]	0.504	3.4	0.153
$MNPs_{cit}$[2]	0.73	1.45	0.69×10^{-4}
$MNPs_{gly}$[2]	0.20	2.97	1.41×10^{-4}
$MNPs_{PLL}$[2]	0.19	4.50	2.14×10^{-4}
$MNPs_{PEI}$[2]	0.12	6.10	2.90×10^{-4}
NH_2-MION[3]	0.22	6.2	-
SH_2–NH_2-MION[3]	0.25	6.0	-
AuNP-hybrid[4]	440	0.734	-
V_2O_5 nanowires[5]	0.004	-	2.5×10^3
**Nanoparticle**	**TMB**		
	**K_M (mmol/L)**	**V_{max} (10^{-7} M/s)**	**k_{cat} (s^{-1})**
Mb–MNPs[1]	**0.504**		
Fe_3O_4 MNPs[6]	0.098	0.34	3.02×10^4
HRP[6]	0.434	1	4.00×10^3
Co_3O_4[7]	0.037	0.63	1.83×10^2
Pt–Ft[8]	0.22	5580	-
PB-MNPs3[9]	0.307	10.6	3.43×10^3
Pt nanoparticle[10]	0.42	0.3	1.78×10^{-3}

Nanoparticle	ABTS		
	K_M (mmol/L)	V_{max} (10^{-7} M/s)	k_{cat} (s^{-1})
H_2TCPP-NiO[11]	0.0114	4.82	-
CuS[12]	0.0072	0.896	-
Co_3O_4[13]	0.063	18.8	376

References.

1. Premaratne, G., Nerimetla, R., Matlock, R., Sunday, L., Koralege, R. S. H., Ramsey, J. D., & Krishnan, S. (2016). Stability, scalability, and reusability of a volume efficient biocatalytic system constructed on magnetic nanoparticles. *Catalysis science & technology*, *6*(7), 2361-2369.
2. Yu, F., Huang, Y., Cole, A. J., & Yang, V. C. (2009). The artificial peroxidase activity of magnetic iron oxide nanoparticles and its application to glucose detection. *Biomaterials*, *30*(27), 4716-4722.
3. Liu, Y., & Yu, F. (2011). Substrate-specific modifications on magnetic iron oxide nanoparticles as an artificial peroxidase for improving sensitivity in glucose detection. *Nanotechnology*, *22*(14), 145704.
4. Boujakhrout, A., Díez, P., Martínez-Ruíz, P., Sánchez, A., Parrado, C., Povedano, E., ... & Villalonga, R. (2016). Gold nanoparticles/silver-bipyridine hybrid nanobelts with tuned peroxidase-like activity. *RSC Advances*, *6*(78), 74957-74960.
5. André, R., Natálio, F., Humanes, M., Leppin, J., Heinze, K., Wever, R., ... & Tremel, W. (2011). V2O5 nanowires with an intrinsic peroxidase-like activity. *Advanced Functional Materials*, *21*(3), 501-509.
6. Gao, L., Zhuang, J., Nie, L., Zhang, J., Zhang, Y., Gu, N., ... & Yan, X. (2007). Intrinsic peroxidase-like activity of ferromagnetic nanoparticles. *Nature nanotechnology*, *2*(9), 577.
7. Mu, J., Wang, Y., Zhao, M., & Zhang, L. (2012). Intrinsic peroxidase-like activity and catalase-like activity of Co 3 O 4 nanoparticles. *Chemical Communications*, *48*(19), 2540-2542.
8. Fan, J., Yin, J. J., Ning, B., Wu, X., Hu, Y., Ferrari, M., ... & Nie, G. (2011). Direct evidence for catalase and peroxidase activities of ferritin–platinum nanoparticles. *Biomaterials*, *32*(6), 1611-1618.
9. Zhang, X. Q., Gong, S. W., Zhang, Y., Yang, T., Wang, C. Y., & Gu, N. (2010). Prussian blue modified iron oxide magnetic nanoparticles and their high peroxidase-like activity. *Journal of Materials Chemistry*, *20*(24), 5110-5116.
10. Park, J. M., Jung, H. W., Chang, Y. W., Kim, H. S., Kang, M. J., & Pyun, J. C. (2015). Chemiluminescence lateral flow immunoassay based on Pt nanoparticle with peroxidase activity. *Analytica chimica acta*, *853*, 360-367.
11. Liu, Q., Yang, Y., Li, H., Zhu, R., Shao, Q., Yang, S., & Xu, J. (2015). NiO nanoparticles modified with 5, 10, 15, 20-tetrakis (4-carboxyl pheyl)-porphyrin: Promising peroxidase mimetics for H2O2 and glucose detection. Biosensors and Bioelectronics, 64, 147-153.
12. Dutta, A. K., Das, S., Samanta, S., Samanta, P. K., Adhikary, B., & Biswas, P. (2013). CuS nanoparticles as a mimic peroxidase for colorimetric estimation of human blood glucose level. *Talanta*, *107*, 361-367.
13. Jia, H., Yang, D., Han, X., Cai, J., Liu, H., & He, W. (2016). Peroxidase-like activity of the Co 3 O 4 nanoparticles used for biodetection and evaluation of antioxidant behavior. *Nanoscale*, *8*(11), 5938-5945.
